# Effects of online learning climate on students’ engagement and wellbeing: the mediating role of teacher credibility and positive academic emotions

**DOI:** 10.3389/fpsyg.2026.1861220

**Published:** 2026-07-24

**Authors:** Yafei Shi, Jiali Chen, Yantao Wei, Mingyue Wu, Xiang Ji, Ke Zhu

**Affiliations:** 1 Faculty of Education, Henan Normal University, Xinxiang, China; 2 Henan Collaborative Innovation Center for Intelligent Education, Henan Normal University, Xinxiang, China; 3 Hubei Key Laboratory of Digital Education, Central China Normal University, Wuhan, China; 4 School of Humanities and Law, Anyang University, Xinxiang, China

**Keywords:** control-value theory, learning engagement, online learning climate, positive academic emotions, social cognitive theory, teacher credibility, wellbeing

## Abstract

**Introduction:**

Learning engagement and wellbeing are critical determinants of the quality of online education and students’ physical and mental health. However, the mechanism that affects online learning engagement and wellbeing is unclear. Therefore, the present study aimed to explore the factors influencing learning engagement and wellbeing in an online environment as well as the mechanisms of their interaction.

**Methods:**

The study collected data from 343 graduate students at a university in central China and employed partial least squares structural equation modeling for analysis.

**Results:**

Online learning climate positively predicted teacher credibility and positive academic emotions, while its direct effects on learning engagement and wellbeing were not significant. Teacher credibility mediated the effect of online learning climate on both learning engagement and wellbeing. While positive academic emotions acted as a mediator in association between online learning climate and learning engagement, no significant mediating effect of positive academic emotions was found for the relationship between online learning climate and wellbeing. In addition, teacher credibility and positive academic emotions sequentially mediated the association between the online learning climate and learning engagement.

**Discussion:**

This study highlights teacher credibility as a central mediating mechanism linking online learning climate to student engagement and wellbeing, thereby advancing theoretical understanding of psychological processes in online learning contexts. Practically, it underscores the importance of online teacher professional development in enhancing educators’ expertise, trustworthiness, and likeability to foster positive student outcomes.

## 1 Introduction

Over the past several years, online learning has increasingly captured the attention of researchers. The global COVID-19 pandemic has additionally propelled the popularization and development of online education (Barrot et al., 2021; Qi et al., 2025). Compared with traditional learning methods, the advantages of online learning, such as flexible time and space options, on-demand access, and massive learning resources, can effectively meet the educational needs of lifelong education, blended learning, and personalized learning (Castro and Tumibay, 2021; Zeng and Luo, 2024). However, the spatial separation between teachers and students in online courses renders student engagement ambiguous and difficult to measure (Cole et al., 2021). Furthermore, the absence of humanizing factors in technological environments may engender feelings of isolation among students (Dumford and Miller, 2018), resulting in diminished learning motivation, inadequate engagement levels, and lower completion rates in online learning contexts. Learning engagement (LEE) represents the positive and sustained psychological state demonstrated by learners throughout the learning process (An et al., 2024). It serves as a vital indicator of online education quality and plays a important role in fostering active learning (Cui and Wang, 2024; Wang et al., 2022). In addition, an increasing number of researchers are acknowledging that the aim of education should not only be to obtain a single cognitive outcome, but also focus on non-cognitive factors of students, such as developing wellbeing (WB) (Jackman et al., 2025). WB serves not only as a critical metric for assessing educational quality (Zheng, 2022), but also exhibits a strong correlation with students’ life quality, mental, and physical health (Terzioglu et al., 2024). Consequently, considering the significance of LEE and WB, this study seeks to investigate the impact of the online learning environment on students’ LEE and WB, while elucidating the underlying psychological mechanisms.

From the perspective of social cognitive theory (SCT), environmental factors (e.g., learning climate and teacher factors) and personal factors (e.g., motivation, academic emotions, and self-efficacy) and the interactions between these factors affect individuals’ perceived behavior (e.g., LEE and WB) (Bandura, 1999; Cai and Shi, 2022; Sheu et al., 2017; Wang and Zhou, 2025; You and Kang, 2014; Zhang, 2009). Online learning climate (OLC) involves associations and interactions among instructors, students, and courses (Bravo-Sanzana et al., 2024), serving as a critical determinant of online LEE, student WB, and instructional quality (Cao, 2025; Wang et al., 2020). Understanding the underlying influence mechanism of OLC on LEE and WB offers significant theoretical insights and practical guidance for developing online learning environments that foster high levels of engagement and learner satisfaction. Teachers, as a vital aspect in learning environments, not only have a substantial impact on students’ performance but also contribute to students’ WB (Zhang, 2009; Zheng, 2022). Teacher credibility (TEC), a critical attribute of teachers in the teaching and learning process, exerts a positive influence on learners’ motivation, satisfaction, and LEE (Froment and de-Besa Gutiérrez, 2022). Exploring the links between TEC, LEE, and WB holds substantial value for researchers and practitioners seeking to implement effective strategies to enhance learning outcomes and learner experience in online contexts (Zheng, 2022; Zheng, 2021b). Nevertheless, empirical research examining the association between TEC and WB remains notably scarce. Academic emotion refers to a variety of emotional experiences encountered by students within the learning and teaching process, including hope, pride, enjoyment, boredom, and anxiety etc. (Pekrun et al., 2002). The generation of these emotions significantly influences students’ motivation, learning strategy use, academic performance, and overall academic achievement (Lei et al., 2018; You and Kang, 2014). Existing literature indicates that positive academic emotions (PAE) contribute to LEE and WB (Bastian et al., 2014; Zhen et al., 2017). However, the specific roles of TEC and PAE in mediating the impact of the OLC on students’ LEE and WB remain underexplored.

To address existing research gaps, this study constructed a multiple mediator model aimed at exploring the effects of OLC, TEC, and PAE on LEE and WB, as well as the influencing mechanism from OLC to LEE and WB. This study contributes to implications for online education practice and the construction of online learning environments.

## 2 Theoretical framework

### 2.1 Social cognitive theory

SCT has found broad application in education, psychology, and health sciences (Schunk and DiBenedetto, 2020). SCT focuses on the dynamic, bidirectional interactions between personal factors, behavioral patterns, and environmental events (Bandura, 1999). Bandura (1986) posits that human behavior operates within social contexts, shaped by the interplay personal factors and environmental conditions. Specifically, environmental factors usually include social environment, climate, learning environment, and interpersonal relationships (Bandura, 2001; Cai and Shi, 2022; Leiter and Maslach, 1988; Shahzad et al., 2025). Personal factors typically encompass ability, expectation, attitude, emotion, motivation, and self-efficacy (Bandura, 1999; Schunk and Usher, 2012). Behavioral factors commonly involve learning behaviors, health behaviors, and behavioral outcomes (Bandura, 2001; Herbert et al., 2021; Limniou et al., 2021). For instance, in order to examine how LEE shapes perceived learning outcomes, Panigrahi et al. (2021) first investigated the effects of personal factors (i.e., internet self-efficacy) and online learning environmental factors (i.e., the quality of information, systems, and services) on students’ engagement behaviors. Subsequently, they explored whether engagement in turn predicts online learning effectiveness, finding that it fully mediates the link between online self-efficacy and perceived learning effectiveness. Similarly, Herbert et al. (2021) explored the relationship among the COVID-19 pandemic (social environment), emotions, personality (personal factors), and health behaviors, work behaviors, and study behaviors (behaviors factors) through linguistic analysis, correlation analysis, and machine learning. Their findings revealed a notable rise in college students’ perceived emotional struggles, academic behavioral challenges, and physical health concerns during the pandemic. However, current research predominantly focuses on cognitive outcomes, such as LEE and academic performance (Hosen et al., 2021; Panigrahi et al., 2021), with relatively less attention devoted to student WB a mental health behavioral outcome. Therefore, this study seeks to examine the factors shaping learning behaviors and mental health outcomes, as well as their underlying psychological mechanisms within a unified framework in online learning settings. Specifically, the perceived learning climate and TEC in the online learning process are considered environmental factors, while students’ PAE are regarded as personal factors.

### 2.2 Control-value theory

CVT is a psychological theory that investigates the interplay between achievement emotions, their causes, and their consequences (Pekrun, 2006). Achievement emotions are characterized as feelings associated with achievement activities or outcomes, such as those caused by academic success or failure (Pekrun et al., 2007). The present study focuses on the former, specifically PAE elicited by online learning activities. Pekrun and Perry (2014) assert that appraisals are critical to the arousal of emotions, especially control appraisals and value appraisals. Perceived value encompasses both the perceived significance (goal relevance) and orientation (consistency with or hindrance to goal attainment) (Pekrun and Perry, 2014). Drawing on rhetorical/relational goal theory, teacher interpersonal behaviors, such as TEC, facilitate students’ achievement of academic and relational goals (Lv, 2024; Myers et al., 2018). Accordingly, the present study employed students’ perceptions of TEC as perceived value. Achievement emotions are prevalent in educational contexts, where they regulate and intervene in student performance and mental health (Pekrun and Perry, 2014). Thus, this study focuses on the impact of achievement emotions, specifically PAE, on LEE and WB.

In summary, drawing on SCT and CVT, this study seeks to explore the influence of OLC on LEE and WB, while also examining whether TEC and PAE act as mediators in these relationships.

## 3 Literature review and hypothesis development

### 3.1 Learning engagement

In educational contexts, engagement refers to the duration and intensity level of a learner’s thinking, acting, and feeling in learning activities (Lu and Churchill, 2014). LEE is conceptualized as a meta-construct comprising multiple dimensions, including behavioral, cognitive, and emotional engagement (Fredricks and McColskey, 2012). Reeve and Tseng (2011) propose that agentic engagement should be considered as the fourth dimension of LEE, which provides a more comprehensive explanation of how students engage in student activities. In this study, behavioral engagement is defined as students’ active participation in the online learning setting, evidenced by positive behavior, effort, attention, and persistence (Ladd and Dinella, 2009). Emotional engagement refers to learners’ emotional responses to online classes, including the presence of interest, enthusiasm, and fun, as well as the absence of anger, anxiety, and boredom (Maricic and Lavicza, 2024). Cognitive engagement involves the cognitive effort exerted to grasp complex concepts and master challenging skills, which includes the use of both deep and shallow learning strategies (Fredricks et al., 2004; Li and Lajoie, 2022). Agentic engagement represents the constructive contributions students make to the instructional process, such as taking the initiative to ask the teacher questions (Reeve and Tseng, 2011). Student engagement is the cornerstone of high-quality development of education and a sign that students play an active role (Fisher et al., 2021; Reeve and Tseng, 2011). Moreover, it is a critical determinant of academic success, learning satisfaction, learning outcomes, and learning persistence (Fisher et al., 2021; Hu and Hui, 2012; Jung and Lee, 2018).

### 3.2 Wellbeing

WB is the ultimate motivation for human behavior (Diener, 1984) and a key indicator for assessing quality of education (Zheng, 2022). It is a broad concept that can be measured by objective or subjective indicators (Kaya and Erdem, 2021; Sonnentag, 2015). In this study, student WB refers to subjective wellbeing, conceptualized as an individual student’s cognitive evaluation of their life and emotions, such as perceptions of meaning, interest, and satisfaction in life. Research posits that if schools promote positive, healthy, and effective learning environments, the WB of students during the learning process must not be overlooked (Currie, 2020). Moreover, Subjective wellbeing, in particular, is closely related to student’s academic achievement (Kaya and Erdem, 2021). Assessing subjective wellbeing not only contributes to the effective achievement of expected educational goals but also promotes students’ physical health and reduces psychological problems (Renshaw et al., 2024). However, schools currently tend to prioritize students’ academic outcomes and career objectives, often neglecting the critical importance of student WB (Graham et al., 2016).

### 3.3 Online learning climate

As defined by Kaufmann et al. (2016), OLC describes the sense of connection, rapport, or affinity perceived by instructors and students in online learning environments, which consists of course clarity, course structure, student connectedness, and instructor behavior. Specifically, instructor behavior refers to the supportive, empathetic, and responsive actions of instructors in online communication (Kaufmann et al., 2016). Course clarity is described as the clearness with which the teacher communicates class content (Kaufmann and Vallade, 2022). Course structure is associated with learners’ perceived opportunities for interaction and involvement in online learning environments (Kaufmann and Vallade, 2022). Student connectedness reflects opportunities for interaction with peers (Kaufmann et al., 2016). Studies consistently show that classroom climate plays a significant role in shaping learners’ learning outcomes (Luo and Derakhshan, 2024). Positive classroom climates contribute to increased learner satisfaction, motivation, classroom engagement, and overall WB, while alleviating negative emotions such as anxiety and fear (Qiu, 2022; Wang et al., 2020).

Existing studies have identified a close association between elements of learning climate and TEC (Finn et al., 2009; Yu et al., 2021; Zheng, 2021b). For example, Yu et al. (2021) explored the relationship between self-regulated learning, TEC, information and communication technology literacy, and academic performance in blended learning contexts. They suggested that teachers can improve teacher-student relationships by expressing respect, care, and warmth toward students, thereby shaping a broader learning climate that further influences students’ perceptions of TEC. Similarly, Finn et al. (2009) explored the links between TEC, instructor behavior, and student academic outcomes, suggesting TEC as a potential mediating factor of the relationship between teacher behavior and student outcomes. Likewise, Zheng (2021a) explored the effects of TEC and teacher clarity on willingness to attend classes and student’ engagement, revealing robust associations among teacher immediacy, TEC, and teacher clarity. These findings indicate that teachers can strengthen the learning climate through improved their behavior, course clarity, which in turn improves students’ perceptions of TEC. From the above analysis, we can see that although existing work have explored connections between specific learning climate elements and TEC, few studies have examined the relationship between overall learning climate variables and TEC. Additionally, extensive research demonstrates that supportive classroom climates in traditional learning environments influences students’ positive emotions, LEE, and WB (Goagoses et al., 2024; Reyes et al., 2012; Tang et al., 2021; Wang et al., 2020). For example, Tang et al. (2021) utilized a multiple mediation model to examine the roles of self-efficacy and achievement emotions in the relationship between learning climate and persistence. Their findings indicated that learning climate was positively associated with persistence, self-efficacy, and positive achievement emotions (enjoyment). Reyes et al. (2012) surveyed 1,399 fifth-grade and sixth-grade students, revealing that higher classroom emotional climate was linked to increased engagement and academic achievement. Similarly, Wang et al. (2020) concluded that classroom climate is a critical factor in promoting children’s academic and psychological WB and improving school quality. As a result, we made the following hypotheses:

*H1*: OLC positively influences TEC (a), PAE (b), LEE (c), and WB (d).

### 3.4 Teacher credibility

The definition of TEC remains without a unified, clear consensus. Mazer et al. (2009) conceptualize TEC as students’ perceptions of an instructor’s competence, caring, and trustworthiness. Little and Green (2022) describe credibility in education as encompassing trustworthiness, expertise, and identification. Sia et al. (2024) define teacher credibility as expertise, credibility, and likeability based on source credibility theory. In this context, Expertise is the skills or knowledge required to achieve teaching objectives; trustworthiness denotes the extent to which teachers convey knowledge persuasively and reliably; and likeability reflects the degree to which a teacher possesses qualities that are liked by students, such as friendliness, warmth, and approachability (Sia et al., 2024).

TEC is a decisive factor in teachers’ ability to effectively influence students (Zheng, 2021b). Established studies have shown that teacher credibility positively impacts LEE (Froment and de-Besa Gutiérrez, 2022; Wang and Kruk, 2024). For instance, Wang and Kruk (2024) used a sequential mixed-methods approach, found that both teacher confirmation and credibility positively forecasted LEE among Chinese English-major students, concluding that TEC fosters positive emotions, thereby enhancing students’ activity in the classroom. Another study highlights that educators who possess knowledge, competence, and rapport with learners play a significant role in student development (Zheng, 2022). Meanwhile, learners’ perceived teacher interpersonal behaviors (e.g., teacher credibility) can serve as a key predictor of student WB. Thus, TEC seems to have promising effects on PAE, LEE, and WB. Along this line, we hypothesized that:

*H2*: TEC positively influences PAE (a), student engagement (b), and WB.

### 3.5 Positive academic emotions

Pekrun et al. (2002) framed academic emotions as emotions associated with academic achievement, student learning, and classroom instruction, which are elicited during the teaching and learning process. They viewed valence, arousal and object focus as salient features of emotions, with valence categorizing emotions into two broad types: positive emotions (e.g., pride, relaxation, enjoyment) and negative emotions (e.g., shame, boredom, anxiety) (Pekrun et al., 2023). Negative emotions disrupt students’ attention and hinder academic performance, LEE, and motivation (Deng et al., 2022). Conversely, positive emotions positively affect cognitive learning processes, including attention, cognitive interest, motivation, and engagement (Li et al., 2020; Williams et al., 2013). From a neuroscience perspective, Li et al. (2020) elucidate how positive emotions improve attention, enhance memory, generate appropriate learning motivation and effective learning strategies through the activity of brain regions, while negative emotions exert a counterproductive effect. Williams et al. (2013) surveyed hospitality and business undergraduate students at a U. S. university, finding that positive emotional experiences in the classroom helped to increase students’ motivation levels as well as attenuate emotional exhaustion.

Extensive empirical evidence demonstrates that PAE can positively impact students’ LEE and WB (An et al., 2023; Bastian et al., 2014; Sadoughi and Hejazi, 2021; Zhen et al., 2017). For example, Sadoughi and Hejazi (2021) explored the mediating role of PAE in the relationship between teacher support and academic engagement, demonstrating that teacher support enhances LEE by mobilizing students’ positive emotions. An et al. (2023) investigated 497 students in three secondary schools in East China, revealing that teacher support promoted LEE by stimulating positive emotions, which subsequently enhanced academic performance. Bastian et al. (2014) argued that positive emotions are also closely linked to subjective wellbeing. They analyzed data from 9,000 university students across 47 regions in various countries and found that individuals in cultures valuing positive emotions report higher life satisfaction. Given the literature review, we proposed:

*H3*: PAE positively influence LEE (a) as well as WB (b).

Drawing on these analyses, we found that OLC exerts a positive effect on TEC and PAE (Finn et al., 2009; Reyes et al., 2012; Wang et al., 2020; Yu et al., 2021; Zheng, 2021b). In turn, these two factors have been shown to enhance both LEE and WB (An et al., 2023; Bastian et al., 2014; Wang and Kruk, 2024; Zheng, 2022). Consequently, we infer that TEC and PAE serve as mediators in the relationship between the OLC and both LEE and WB. In addition, a significant association exists between TEC and PAE (Wang and Kruk, 2024). Therefore, we hypothesized that TEC and PAE seem to form a chain of mediators influencing the role of OLC on LEE and WB. The above reasoning leads the following hypothesis:

*H4*: TEC functions as a mediator linking the OLC to LEE (a) and WB (b).

*H5*: PAE function as mediators linking the OLC to LEE (a) and WB (b).

*H6*: TEC and PAE as sequential mediators linking the OLC to LEE (a) and WB (b).

### 3.6 Current study

**Figure 1:**
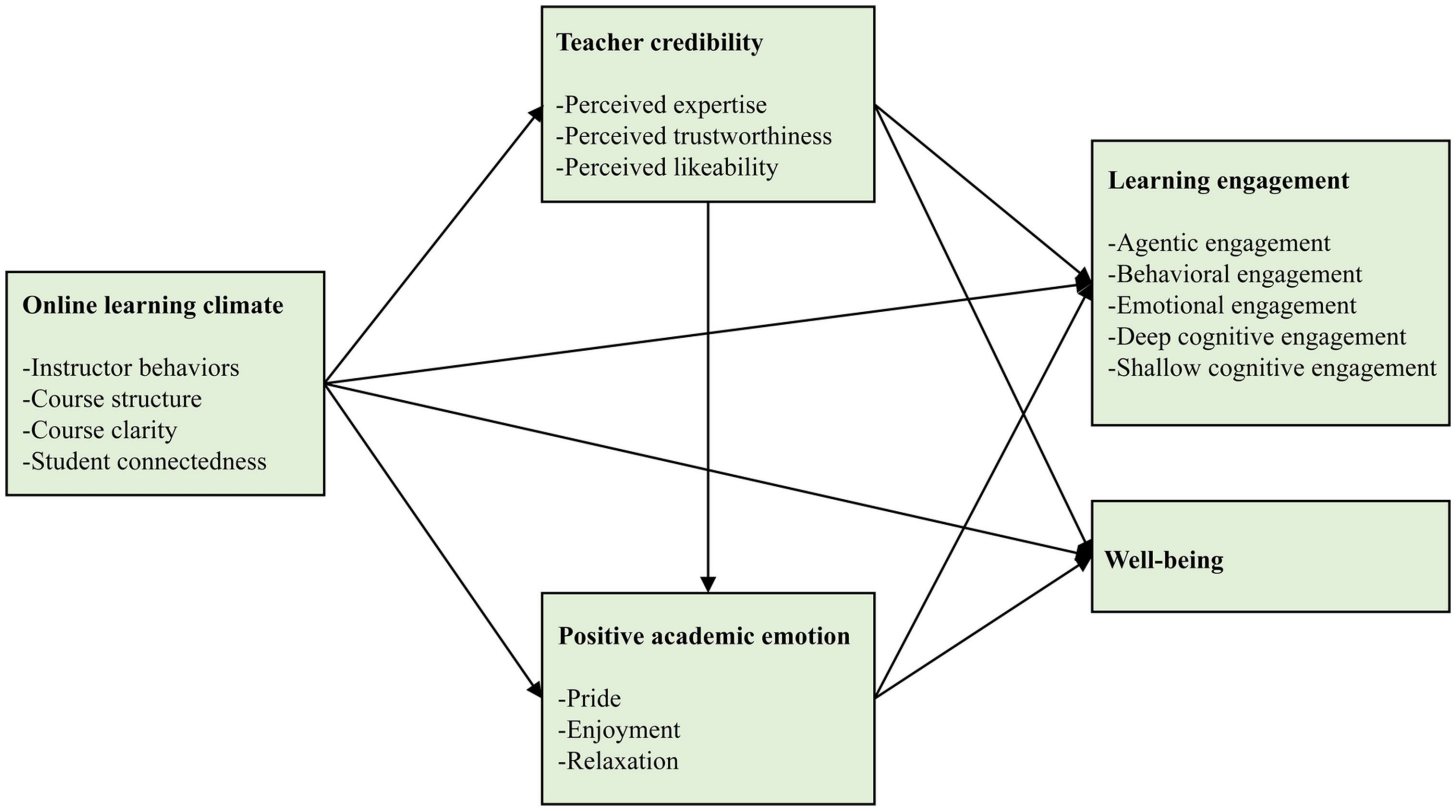
Hypothetical model.

This study aims to examine the impact of OLC on LEE and WB, while further analyzing the potential mediating role of TEC and PAE in the relationships between these variables, thereby elucidating the underlying mechanisms in greater depth. To this end, this study draws on SCT and CVT to constructs a sequential multiple mediation model, as depicted in Figure 1.

## 4 Methodology

### 4.1 Participants

This study used convenience sampling to investigate the online learning experiences of graduate students at a normal university in central Henan Province. Prior to data collection, approval was obtained from relevant administrators, and informed consent was secured from participants, with the study’s purpose clearly communicated to them. All participants completed the survey voluntarily and anonymously, yielding 343 initial responses. After deleting the invalid data, 312 questionnaires were obtained, representing an effective response rate of 91.0%. The participants had an average age of 23 years. The sample comprised 44.6% male students (*n* = 139) and 55.4% female students (*n* = 173). The students whose registered residence is in rural areas account for 66.0% (*n* = 206) and urban areas account for 34.0% (*n* = 106). The distribution of students across graduate years was as follows: 52.6% (*n* = 164) in their first year, 41.7% (*n* = 130) in their second year, and 5.8% (*n* = 18) in their third year. The proportion of students ranking above average, at the average level, and below average in the class was 42.3% (*n* = 132), 42.6% (*n* = 133), and 15.1% (*n* = 47), respectively.

### 4.2 Instruments

The questionnaire was structured into two main sections. The first section collected demographic data from participants, while the second section assessed students’ perceptions of the following constructs: OLC, PAE, TEC, LEE, and WB.

In the present study, all constructs were measured using Likert-type scales. Although individual Likert-type items are generally considered ordinal, composite scores derived from multiple items are widely regarded as approximating interval-level measurement in social science research. Therefore, following established methodological conventions, the present study treated the composite scores of the multi-item constructs as interval-level data suitable for structural equation modeling. This approach has been widely supported in prior methodological literature (e.g., Carifio and Perla, 2008; Norman, 2010).

#### 4.2.1 Online Learning Climate Scale

The OLC was measured using an adapted version of the Online Classroom Climate Scale (OLCS) developed by Kaufmann et al. (2016). This scale consists of four main dimensions: student-perceived course structure (3 items), course clarity (3 items), instructor behavior (5 items), and student connectedness (3 items). The OLCS utilizes a seven-point Likert scale to measure student-perceived OLC, ranging from 1 (strongly disagree) to 7 (strongly agree).

#### 4.2.2 Teacher Credibility Scale

TEC was assessed using the Teacher Credibility Scale developed by Sia et al. (2024). The scale measures students’ perceived TEC across three dimensions, including expertise (4 items), teacher likeability (3 items), and trustworthiness (4 items). Responses are scored on a seven-point Likert scale, (1 = strongly disagree, 7 = strongly agree), with higher scores indicating greater perceived TEC.

#### 4.2.3 Positive Academic Emotions Scale

PAE were primarily measured using the Academic Emotions Questionnaire (AEQ) by Chen (2024). The AEQ uses a five-point Likert scale to assess students’ perceptions of PAE, ranging from 1 (strongly disagree) to 5 (strongly agree). This study focused on three emotions: pride (3 items), enjoyment (3 items), and relaxation (3 items).

#### 4.2.4 Learning Engagement Scale

The LEE scale encompasses five sub-dimensions. Agentic engagement (4 items), behavioral engagement (3 items), and emotional engagement (3 items) scales were adapted from Reeve and Tseng (2011) and measured using a seven-point Likert scale ranging from 1 (strongly disagree) to 7 (strongly agree), with higher scores reflecting greater student engagement. Cognitive engagement c was divided into deep cognitive engagement (5 questions) and shallow cognitive engagement (3 items), adapted from Miller et al. (1996), and assessed using a five-point Likert scale (1 = strongly disagree, 5 = strongly agree) to gauge students’ online cognitive engagement.

#### 4.2.5 Wellbeing Scale

WB construct was measured using the Flourishing Scale of Diener et al. (2010). This scale includes five items, such as “My life is purposeful and full of meaning”. Participants rated their WB on a seven-point Likert scale (1 = strongly disagree, 7 = strongly agree), with higher scores indicating greater levels of WB.

### 4.3 Data collection and analysis

This study collected data on students’ online learning experience through the online questionnaire distribution platform Wenjuanxing.^1^ Initially, the researchers translated the measurement scale into Chinese and entered the questions into the Wenjuanxing platform. Questionnaire distribution was facilitated by a member of the research team, who also served as an instructor at the participants’ institution. Data analysis was conducted using SPSS 25 and SmartPLS3 statistical software. The process began with data cleaning to eliminate invalid responses, followed by confirmatory factor analysis (CFA) to ensure indicator reliability. Partial least squares structural equation modeling (PLS-SEM), which integrates principal component analysis and regression path analysis, is particularly well-suited for complex models and smaller sample sizes compared to covariance-based structural equation modeling (CB-SEM) (Hair et al., 2021). Therefore, the study used PLS-SEM to estimate the measurement and structural model, thereby examining the influence patterns of OLC on LEE and WB.

## 5 Results

### 5.1 Assessment of the measurement model

In this study, the constructs of OLC, PAE, TEC, and LEE are conceptualized as second-order factor structures. To assess the higher-order factor structure, the two-stage approach was used to evaluate the measurement model (Sarstedt et al., 2019). Reliability and convergent validity were evaluated via factor loadings, composite reliability (CR), Cronbach’s alpha, and average variance extracted (AVE), while discriminant validity was assessed via the Fornell-Larcker criterion.

First, the results of the first-stage measurement model evaluation are presented in Supplementary Table S1. All indicator factor loadings were greater than 0.7 (Hair et al., 2012). Cronbach’s *α* values (0.933–0.979) and the CR values (0.957–0.984) both exceeded the recommended criterion of 0.7, and AVE values (0.881–0.953) met the 0.5 threshold indicating robust internal consistency and convergent validity (Bagozzi and Yi, 1988). Table 1 further confirms discriminant validity, as the correlation coefficients of all low-order constructs are lower than the square root of the corresponding AVE (Fornell and Larcker, 1981).

**Table 1:** Discriminant validity of the first stage.

Constructs	IB	CS	CC	SC	PE	PT	PL	PR	EN	RE	AE	BE	EE	DCE	SCE	WB
IB	0.961															
CS	0.948	0.964														
CC	0.937	0.961	0.967													
SC	0.936	0.958	0.964	0.962												
PE	0.888	0.902	0.921	0.919	0.960											
PT	0.905	0.906	0.917	0.920	0.952	0.955										
PL	0.857	0.865	0.872	0.882	0.908	0.930	0.976									
PR	0.865	0.899	0.915	0.904	0.878	0.871	0.819	0.952								
EN	0.822	0.881	0.875	0.881	0.877	0.859	0.827	0.922	0.958							
RE	0.807	0.842	0.834	0.840	0.845	0.828	0.792	0.885	0.895	0.939						
AE	0.816	0.830	0.826	0.826	0.839	0.858	0.869	0.818	0.800	0.809	0.941					
BE	0.853	0.871	0.873	0.876	0.894	0.909	0.928	0.860	0.842	0.831	0.928	0.974				
EE	0.836	0.851	0.848	0.856	0.837	0.851	0.867	0.842	0.833	0.829	0.907	0.920	0.960			
DCE	0.833	0.845	0.850	0.852	0.847	0.862	0.883	0.877	0.864	0.854	0.902	0.911	0.927	0.944		
SCE	0.805	0.821	0.826	0.842	0.816	0.854	0.864	0.856	0.848	0.827	0.869	0.887	0.901	0.947	0.953	
WB	0.855	0.864	0.852	0.872	0.853	0.872	0.894	0.838	0.824	0.813	0.911	0.926	0.948	0.920	0.903	0.943

Lower triangles are correlation coefficients between factors and diagonal lines are AVE square roots. IB, Instructor Behavior; CS, Course Structure; CC, Course Clarity; SC, Student Connectedness; PR, Pride; EN, Enjoyment; RE, Relaxation; PE, Professional Expertise; PT, Credibility; PL, Likeability; AE, Agentic Engagement; BE, Behavioral Engagement; EE, Emotional Engagement; DCE, Deep Cognitive Engagement; SCE, Shallow Cognitive Engagement; WB, Wellbeing.

Subsequently, the estimated scores of the lower-order factors were used to calculate the higher-order factors. The evaluation results of the second-stage measurement model are shown in Table 2. Again, all indicator loadings, Cronbach’s α, AVE, and CR values exceeded their respective thresholds, confirming the reliability and validity of the higher-order structure. Table 3 illustrates the discriminant validity of the second-stage measurement model, with results indicating robust discriminant validity.

**Table 2:** Reliability and convergent validity of the second stage.

Constructs		Loadings	STDEV	*t*	*p*	Cronbach’s ɑ	CR	AVE
OLC	IB	0.973	0.006	153.985	***	0.987	0.991	0.963
	CS	0.985	0.004	266.808	***			
	CC	0.984	0.004	277.267	***			
	SC	0.983	0.004	243.758	***			
TEC	PE	0.976	0.006	159.455	***	0.976	0.984	0.953
	PT	0.984	0.005	211.861	***			
	PL	0.969	0.014	70.100	***			
PAE	PR	0.969	0.006	163.597	***	0.965	0.977	0.934
	EN	0.972	0.005	188.912	***			
	RE	0.958	0.009	102.275	***			
LEE	AE	0.956	0.012	77.188	***	0.981	0.985	0.928
	BE	0.965	0.007	145.504	***			
	EE	0.966	0.006	149.728	***			
	DCE	0.973	0.004	237.874	***			
	SCE	0.956	0.009	104.756	***			
WB	WB3	0.946	0.019	50.309	***	0.969	0.975	0.888
	WB5	0.951	0.010	95.256	***			
	WB6	0.942	0.012	80.053	***			
	WB7	0.948	0.012	75.872	***			
	WB8	0.926	0.021	43.918	***			

***p < 0.001. OLC, online learning climate; PAE, positive academic emotions; TEC, teacher credibility; LEE, learning engagement; WB, wellbeing.

**Table 3:** Discriminant validity of the second stage.

Construct	OLC	TEC	PAE	LEE	WB
OLC	0.981				
TEC	0.935	0.976			
PAE	0.911	0.895	0.966		
LEE	0.891	0.920	0.902	0.963	
WB	0.877	0.894	0.854	0.957	0.943

Lower triangles are inter-factor correlation coefficients and diagonals are AVE square roots. OLC, online learning climate; PAE, positive academic emotions; TEC, teacher credibility; LEE, learning engagement; WB, wellbeing.

### 5.2 Assessment of structural model

In this study, the path coefficients, effect size (f^2^), the coefficient of determination (R^2^), and predictive relevance (Q^2^) were used to assess the structural model. The results are presented in Table 4, the *R*^2^-values of the variables range from 0.818 to 0.878, all of which are greater than 0.75 (Hair et al., 2012), which means that the model explains a substantial proportion of variance in the endogenous variables. Additionally, all *Q*^2^-values were greater than 0 (Hair et al., 2012), indicating adequate predictive relevance for the structural model.

**Table 4:** Assessment of structural model.

Path	*β*	STDEV	*t*	*p*	Confidence intervals		*f* ^2^	*R* ^2^	*Q* ^2^
					2.50%	97.50%			
OLC → TEC	0.935	0.014	67.174	***	0.905	0.959	7.007	0.875	0.826
OLC → PAE	0.598	0.103	5.821	***	0.387	0.787	0.288	0.845	0.783
TEC → PAE	0.335	0.104	3.215	**	0.142	0.546	0.091		
OLC → LEE	0.005	0.122	0.041	0.967	−0.247	0.227	0.000	0.878	0.807
TEC → LEE	0.565	0.149	3.787	***	0.276	0.864	0.299		
PAE → LEE	0.391	0.093	4.216	***	0.217	0.577	0.194		
OLC → WB	0.212	0.143	1.476	0.140	−0.072	0.482	0.024	0.818	0.719
TEC → WB	0.523	0.162	3.224	**	0.211	0.847	0.172		
PAE → WB	0.193	0.105	1.849	0.065	−0.010	0.404	0.032		

**p < 0.01, ***p < 0.001. OLC, online learning climate; PAE, positive academic emotions; TEC, teacher credibility; LEE, learning engagement; WB, wellbeing.

**Figure 2:**
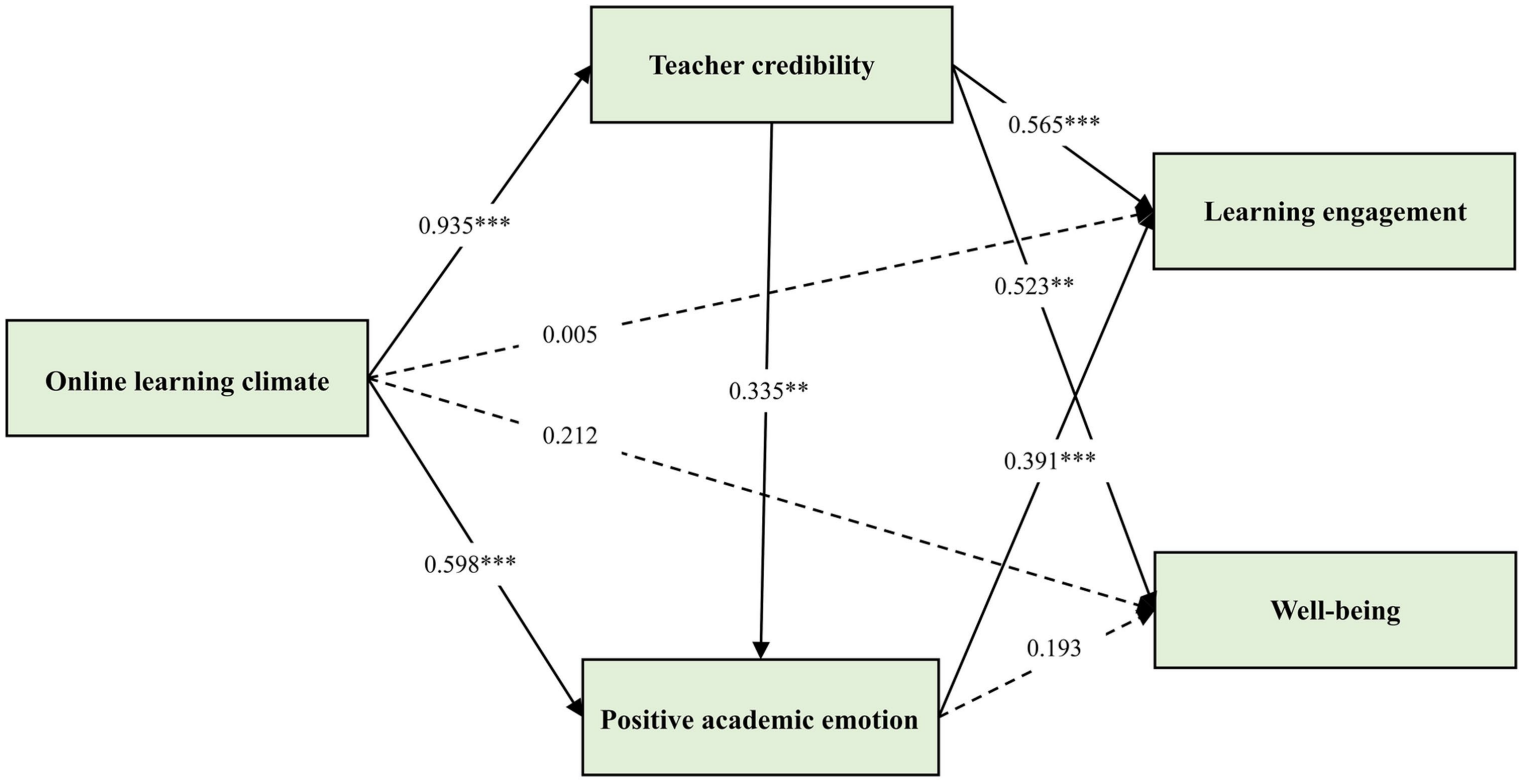
Path analysis results. **p < 0.01, ***p < 0.001.

To test the significance level of the structural model paths, bootstrap sampling with 5,000 iterations was employed (Hair et al., 2011). The results are detailed in Table 4 and Figure 2. The direct effect of OLC on TEC (*β* = 0.935, *p* < 0.001 *f*^2^ = 7.007,) and PAE (*β* = 0.598, *p* < 0.001, *f*^2^ = 0.288) was significant, however, the direct effect on LEE (*β* = 0.005, *p* > 0.05, *f*^2^ = 0.000) and WB (*β* = 0.212, *p* > 0.05, *f*^2^ = 0.024) were not significant. Therefore, H1 is partially valid. TEC exhibited significant positive effect on PAE (*β* = 0.335, *p* < 0.01, *f*^2^ = 0.091), LEE (*β* = 0.565, *p* < 0.001, *f*^2^ = 0.299), and WB (*β* = 0.523, *p* < 0.01, *f*^2^ = 0.172). Therefore, H2 holds true. PAE significantly influenced LEE (*β* = 0.391, *p* < 0.001, *f*^2^ = 0.194), but the effect on WB (*β* = 0.193, *p* > 0.05, *f*^2^ = 0.032) was not significant. Therefore, H3 partially holds.

### 5.3 Mediating role test of positive academic emotions and teacher credibility

As presented in Table 4, most of the direct path effects were significant. Therefore, this study further analyzes the mediating role of TEC and PAE in the relationship between OLC and both LEE and WB. As shown in Table 5, TEC significantly mediated the effect of OLC on LEE (*β* = 0.529, *p* < 0.001, [0.258, 0.813]) and WB (*β* = 0.489, *p* < 0.01, [0.197, 0.792]), thereby fully supporting H4. In addition, PAE significantly mediated the effect of OLC on LEE (*β* = 0.234, *p* < 0.001, [0.117, 0.374]). Therefore, H5 partially holds. Finally, TEC and PAE have a chain mediating role in the influence of OLC on LEE (*β* = 0.123, *p* < 0.05, [0.043, 0.247]). Therefore, H6 is partially established.

**Table 5:** Mediated and total effects analysis.

Path	*β*	STDEV	*t*	*p*	Confidence intervals	
					2.50%	97.50%
Specific indirect effects						
OLC → TEC → LEE	0.529	0.139	3.795	***	0.258	0.813
OLC → PAE → LEE	0.234	0.067	3.493	***	0.117	0.374
OLC → TEC → PAE → LEE	0.123	0.053	2.338	*	0.043	0.247
OLC → TEC → WB	0.489	0.151	3.233	**	0.197	0.792
OLC → PAE → WB	0.116	0.063	1.823	0.068	−0.006	0.246
OLC → TEC → PAE → WB	0.061	0.043	1.417	0.157	−0.003	0.166
Total effects						
OLC → LEE	0.891	0.030	30.037	***	0.824	0.938
OLC → WB	0.877	0.032	27.015	***	0.805	0.929

*p < 0.05, **p < 0.01, ***p < 0.001. OLC, online learning climate; PAE, positive academic emotions; TEC, teacher credibility; LEE, learning engagement; WB, wellbeing.

## 6 Discussion

Grounded in SCT and CVT, this study developed a path model to examine the influence of OLC on LEE and WB, while also testing the mediating roles of TEC and PAE in its influence process.

First, it was found that students perceived OLC positively predicted both TEC and PAE. This indicates that instructors can foster a conducive learning climate by thoughtfully designing course content, actively engaging in teacher-student interactions, and appropriately encouraging collaborative learning among students, thereby enhancing students’ PAE and perceptions of TEC. This finding aligns with Tang et al. (2021), who found that self-efficacy and achievement emotions serve as serial mediators linking learning climate to persistence, with the learning climate positively predicting persistence, self-efficacy, and positive achievement emotions while suppressing negative emotions. Similarly, Finn et al. (2009) reported a moderate correlation between teacher credibility, instructor behaviors, teacher characteristics, and learning outcomes, supporting these findings. Consequently, in online learning environments, instructors should prioritize timely communication, robust course design, clarity, and interactivity to cultivate a favorable learning climate. Notably, however, the direct effects of the OLC on LEE and WB were not supported. This divergence from prior studies (Bravo-Sanzana et al., 2024; Cao, 2025; Goagoses et al., 2024) may be attributable to the limited sense of presence, immediate feedback, and interaction in online settings, which could contribute to reduced levels of engagement and student satisfaction (Cui et al., 2013).

Second, this study revealed that TEC positively influenced students’ PAE, LEE, and WB. That is, teachers with greater expertise, trustworthiness, and likeability shape students’ perceptions of TEC, which subsequently impacts their academic emotions, engagement, and WB. These findings corroborate Lv (2024) observations that TEC fosters positive emotions and promotes LEE. Additionally, the results also align with Zheng (2022) findings that mutual trust, respect, and warmth in teacher-student relationships significantly predict learners’ WB. Furthermore, the current study reveals that TEC acts as a mediating mechanism through which OLC influences both LEE and WB. This suggests that by cultivating a positive OLC, teachers can enhance students’ perceptions of their professional competence, trustworthiness, and approachability, thereby fostering greater online LEE and WB.

In addition, this study found that PAE positively influence LEE. These results align with Oriol-Granado et al. (2017), who demonstrated that “learners” experience of PAE and teacher autonomy support contribute positively to their self-efficacy, academic performance, and student engagement. Similarly, the findings corroborate those of Zhen et al. (2017), who explored the links between basic psychological need satisfaction, self-efficacy, academic emotions, and LEE, revealing that competence and relatedness satisfaction motivated students’ positive emotions, which then promoted their engagement. Additionally, this study identified PAE as a mediator in the relationship between the OLC and LEE. Therefore, teachers should focus on building an online learning environment of mutual respect, rapport, and positive interaction to stimulate learners’ interest in learning and sense of accomplishment, so that they can actively engage and naturally immerse themselves in learning activities. However, the effect of PAE on learners’ WB was not verified in this study, which contrasts with findings by Bastian et al. (2014) and Hamdani (2020). The reason for this may be that the mutual balance of positive and negative emotions or the transient nature of the duration of academic emotions (Fredrickson, 2001), potentially obscuring the impact of PAE on WB. Given that the participants in this study were graduate students, their PAE may have been offset by negative influences, such as academic stress. Therefore, to further reveal the effects of PAE on students’ WB, future studies future research should adopt a more granular approach, employing time-series analyses or intensive sampling methods, such as experience sampling, to examine these dynamics in greater detail.

Interestingly, this study found that the sequential mediation chain comprising TEC and PAE significantly mediates the relationship between the OLC and LEE. This finding aligns with SCT, which posits that students’ perceived environment (learning climate and teacher credibility) influences their behavioral outcomes (learning engagement) through personal factors such as emotions (positive academic emotions) (Bandura, 1999). Specifically, a positive OLC enhances students’ perceptions of TEC. This aligns with the viewpoints of Yu et al. (2021) and Meyers et al. (2006) that conveying respect to students, clarifying course objectives, involving students in problem-solving, and encouraging a sense of classroom community can improve classroom climate and reduce classroom conflict, thereby positively influencing students’ perceptions of teachers and earning their trust. This cognitive appraisal of teacher credibility further promotes students’ academic emotions, consistent with CVT’s assertion that cognitive appraisals elicit emotional responses (Pekrun and Perry, 2014). Ultimately, these positive emotions promote higher LEE, which also confirms the findings of Zhen et al. (2017) and An et al. (2023). These insights suggest that online instructors should focus on the synergistic interplay between students’ perceptions of TEC and PAE to more effectively enhance online learning engagement.

## 7 Implications

### 7.1 Theoretical implications

The study explored the relationship between OLC, TEC, PAE, LEE, and WB. Firstly, this study extends the SCT and CVT to online learning scenarios, not only validating the SCT that behavioral factors (LEE, WB) are affected by environmental factors (OLC, TEC) and personal factors (PAE) and their interactions with each other (Bandura, 1999), but also responding to a key assumption of CVT: that achievement emotions interact with their antecedents and outcomes (Pekrun, 2006). Secondly, the findings demonstrate that the OLC significantly shapes students’ perceptions of TEC, indicating that a teacher’s ability to cultivate a supportive online learning environment influences students’ views of the teacher’s expertise, trustworthiness, and likeability. This result provides a theoretical basis for applying source credibility theory in educational research. Finally, this study identifies two pivotal mediating variables in the online learning environment—PAE and TEC—which collectively form three key pathways through which the OLC enhances learning outcomes or satisfaction: first, by enhancing students’ perceptions of TEC, which promotes both LEE and WB; second, by fostering positive academic emotional experiences, which effectively bolster student engagement; most notably, through the synergistic effect of these two factors, which further facilitates LEE. This discovery sheds light on the dynamic interplay between teacher credibility and positive emotions in online learning environments.

### 7.2 Practical implications

The study also provides some practical guidance for frontline teachers and related practitioners to conduct online teaching. First, the OLC is an important factor that affects TEC and stimulates students’ positive learning emotions. Accordingly, when conducting online teaching, instructors can utilize information technology tools, such as mind maps, which facilitate the organization and presentation of instructional materials while enhancing students’ comprehension and retention of knowledge (Shi et al., 2023), thereby improving course clarity and the rationality of course structure. Additionally, instructors can foster teacher-student and peer-to-peer interactions by incorporating strategies such as group discussions, collaborative tasks, and the establishment of learning communities, thereby cultivating a friendly, positive, and inclusive learning climate. Second, TEC not only exerts a positive impact on learners’ PAE and LEE, but also contributes significantly to their WB. To strengthen students’ perceptions of TEC, instructors should regularly collect feedback, proactively address students’ needs, and make adaptive adjustments to their teaching practices, while actively participating in professional development to continually enhance their expertise and pedagogical skills. Finally, PAE mediate the relationship between OLC and LEE. Consequently, teachers should pay attention to learners’ emotional state when teaching online, design appropriate teaching strategies to compensate for students’ weak motivation and low emotions due to spatial separation. For example, designing gamified teaching to stimulate learners’ PAE’, thereby enhancing their online LEE (Huang and Wang, 2025).

### 7.3 Implications for online teacher professional development

A key finding of this study is that TEC plays a central role in linking the OLC to student learning outcomes. Based on this finding, specific recommendations are proposed for online teacher professional development. These recommendations are organized around the three core dimensions of TEC: expertise, trustworthiness, and likeability.

First, regarding expertise, institutions should provide online teachers with training and resource support in information and communication technology (ICT) and technological pedagogical content knowledge (TPACK). Training should focus on the effective use of digital tools (e.g., artificial intelligence, online course design tools, classroom management tools), electronic resources, online teaching methods, and the ability to address real-time technical issues in online classrooms, thereby enhancing the clarity, structure, and efficiency of knowledge delivery (Dayal, 2023; Khong et al., 2023; Lan, 2024). Furthermore, institutions should establish online teacher learning communities that support teachers in sharing experiences, tips, and resources, helping them demonstrate greater professional competence in online learning environments (Khong et al., 2023). Second, regarding trustworthiness, institutions may encourage online teachers to adopt the following strategies: responding promptly to students’ academic needs to foster perceptions of professional reliability; appropriately sharing personal experiences during instructional interactions and engaging in bounded self-disclosure can convey openness and sincerity to students; and regularly collecting student feedback and adjusting teaching strategies accordingly, which can further enhance students’ perceptions of fairness, reliability, and instructional integrity (Holzer and Daumiller, 2025). Third, regarding likeability, institutions should encourage online teachers to create safe, motivating, inclusive, and equitable learning environments, as well as adopt proactive teaching approaches (Gencoglu et al., 2025). To this end, teachers can be trained to implement student-centered teaching methods (e.g., involving students in the design of instruction and curriculum) (Tsegay et al., 2022). Teachers may also be trained to effectively organize interactive learning activities such as online group discussions, collaborative tasks, and learning communities to enhance students’ social presence (Richardson et al., 2017). In addition, actively listening to student concerns, incorporating engaging activities, and facilitating online extracurricular interactions can help stimulate student motivation and deepen emotional connections (Gencoglu et al., 2025).

## 8 Conclusion and limitations

This study examined the critical factors influencing learners’ LEE and WB in the online learning environment, as well as their underlying relationships. The findings indicated that TEC mediated the impact of OLC on learners’ WB, while TEC and PAE sequentially mediated the positive association between OLC and LEE. These results yield significant implications for the advancement of online learning and the design of effective online learning environments. However, the study has the following limitations. First, the participants comprised just over 300 graduate students from a single normal university in central China, which is a small sample size and a small coverage. Therefore, future research needs to further expand the sample size, collect participants from different academic stages and geographical locations, and improve the generalizability of research conclusions. Second, this study used a cross-sectional design, which neglected the influence of time factors on modeled path relationships. Thus, a longitudinal study design should be used in the future to further validate the stability of the model path relationship. Third, this study relied solely on quantitative data, which constrains the depth of explanation for the relationships among variables. To enhance the interpretability of the findings, future research should incorporate qualitative data collection methods.

## Data Availability

The raw data supporting the conclusions of this article will be made available by the authors, without undue reservation.

## Footnotes

1
www.wjx.cn
