# Supplementary material for: Effects of online learning climate on students’ engagement and wellbeing: the mediating role of teacher credibility and positive academic emotions
Source: Front Psychol. 2026 Jul 24;17:1861220. doi: 10.3389/fpsyg.2026.1861220 (PMC13449235; doi:10.3389/fpsyg.2026.1861220)
Supplement: Supplementary file 1 [file Table_1.DOCX]

**Supplementary Material**

# 1 Supplementary Table

**Supplementary Table S1** Reliability and convergent validity of the first stage

| **Constructs** | | **Loadings** | **STDEV** | ***t*** | ***p*** | **Cronbach’s ɑ** | **CR** | **AVE** |
| --- | --- | --- | --- | --- | --- | --- | --- | --- |
| IB | IB1 | 0.958 | 0.012 | 79.003 | *** | 0.979 | 0.984 | 0.923 |
|  | IB2 | 0.951 | 0.013 | 75.569 | *** |  |  |  |
|  | IB3 | 0.972 | 0.007 | 130.351 | *** |  |  |  |
|  | IB4 | 0.961 | 0.010 | 94.783 | *** |  |  |  |
|  | IB5 | 0.961 | 0.008 | 115.998 | *** |  |  |  |
| CS | CS1 | 0.971 | 0.006 | 161.012 | *** | 0.962 | 0.975 | 0.929 |
|  | CS2 | 0.950 | 0.010 | 92.762 | *** |  |  |  |
|  | CS3 | 0.971 | 0.007 | 131.950 | *** |  |  |  |
| CC | CC1 | 0.975 | 0.006 | 169.496 | *** | 0.965 | 0.977 | 0.934 |
|  | CC2 | 0.951 | 0.012 | 81.458 | *** |  |  |  |
|  | CC3 | 0.973 | 0.007 | 142.446 | *** |  |  |  |
| SC | SC1 | 0.967 | 0.007 | 135.497 | *** | 0.960 | 0.974 | 0.925 |
|  | SC2 | 0.947 | 0.012 | 81.716 | *** |  |  |  |
|  | SC3 | 0.971 | 0.006 | 154.944 | *** |  |  |  |
| PE | PE1 | 0.974 | 0.006 | 174.811 | *** | 0.971 | 0.979 | 0.921 |
|  | PE2 | 0.955 | 0.012 | 78.045 | *** |  |  |  |
|  | PE3 | 0.952 | 0.011 | 83.721 | *** |  |  |  |
|  | PE4 | 0.957 | 0.012 | 82.449 | *** |  |  |  |
| PT | PT1 | 0.963 | 0.009 | 101.469 | *** | 0.968 | 0.977 | 0.912 |
|  | PT2 | 0.943 | 0.011 | 82.708 | *** |  |  |  |
|  | PT3 | 0.956 | 0.010 | 92.926 | *** |  |  |  |
|  | PT4 | 0.958 | 0.011 | 85.048 | *** |  |  |  |
| PL | PL1 | 0.976 | 0.006 | 157.957 | *** | 0.975 | 0.984 | 0.953 |
|  | PL2 | 0.977 | 0.006 | 161.614 | *** |  |  |  |
|  | PL3 | 0.976 | 0.008 | 122.840 | *** |  |  |  |
| PR | PR1 | 0.961 | 0.007 | 131.095 | *** | 0.948 | 0.966 | 0.906 |
|  | PR2 | 0.962 | 0.007 | 130.601 | *** |  |  |  |
|  | PR3 | 0.932 | 0.014 | 66.818 | *** |  |  |  |
| EN | EN1 | 0.956 | 0.009 | 104.808 | *** | 0.955 | 0.971 | 0.917 |
|  | EN2 | 0.963 | 0.008 | 127.530 | *** |  |  |  |
|  | EN3 | 0.953 | 0.012 | 78.075 | *** |  |  |  |
| RE | RE1 | 0.948 | 0.009 | 106.179 | *** | 0.933 | 0.957 | 0.881 |
|  | RE2 | 0.939 | 0.013 | 69.935 | *** |  |  |  |
|  | RE3 | 0.929 | 0.014 | 64.672 | *** |  |  |  |
| AE | AE1 | 0.935 | 0.017 | 53.865 | *** | 0.957 | 0.969 | 0.885 |
|  | AE2 | 0.935 | 0.012 | 77.110 | *** |  |  |  |
|  | AE3 | 0.959 | 0.008 | 118.062 | *** |  |  |  |
|  | AE4 | 0.933 | 0.021 | 44.846 | *** |  |  |  |
| BE | BE1 | 0.970 | 0.008 | 121.671 | *** | 0.973 | 0.982 | 0.949 |
|  | BE2 | 0.972 | 0.008 | 123.032 | *** |  |  |  |
|  | BE3 | 0.980 | 0.005 | 201.556 | *** |  |  |  |
| EE | EE1 | 0.963 | 0.008 | 124.339 | *** | 0.958 | 0.973 | 0.922 |
|  | EE2 | 0.965 | 0.009 | 110.999 | *** |  |  |  |
|  | EE3 | 0.953 | 0.012 | 82.778 | *** |  |  |  |
| DCE | CE1 | 0.942 | 0.013 | 74.345 | *** | 0.969 | 0.976 | 0.891 |
|  | CE2 | 0.957 | 0.008 | 113.787 | *** |  |  |  |
|  | CE3 | 0.912 | 0.016 | 58.506 | *** |  |  |  |
|  | CE4 | 0.957 | 0.008 | 119.202 | *** |  |  |  |
|  | CE5 | 0.950 | 0.009 | 102.221 | *** |  |  |  |
| SCE | CE6 | 0.938 | 0.011 | 83.729 | *** | 0.949 | 0.967 | 0.908 |
|  | CE7 | 0.954 | 0.009 | 103.136 | *** |  |  |  |
|  | CE8 | 0.965 | 0.007 | 140.180 | *** |  |  |  |

Note: ****p*<0.001. IB: Instructor behavior; CS: Course structure; CC: Course clarity; SC: Student connectedness; PR: Pride; EN: Enjoyment; RE: Relaxation; PE: Expertise; PT: Trustworthiness; PL: Likeability; AE: Agentic engagement; BE: Behavioral engagement; EE: Emotional engagement; DCE: Deep cognitive engagement; SCE: Shallow Cognitive Engagement.
